# Autopsy of King Louis of Bavaria

**Published:** 1886-09

**Authors:** 


### Medical News.

### *AUTOPSY OF KING LOUIS OF BAVARIA.*

The results of the autopsy give a key to clinical manifestations, observed in the King during several years past. The lesions of the cranium and brain were especially interesting. The scalp was thick, the cranium small and asymetrical. The vault was extremely thin, the frontal and sagittal sutures ossified. The frontal suture contained osteophytes on the inner surface. The superior longitudinal sinus was greatly dilated posteriorly, and contracted anteriorly near the ethmoid; Pacchionian bodies prominent; dura mater thickened, especially in its frontal portion, and very hyperæmic. The sphenoid and the pterygoid processes contained exostoses; the sella turcica was asymetrical, porous and friable. . The sinuses of the base were engorged with black blood. The weight of the brain was 1,349 grammes (43½ oz.) The arachnoid was thickened. At the summit of the left ascending frontal convolution, in its anterior portion, the arachnoid and pia mater were adherent; in this same place the cranial vault was as thin as a sheet of paper. Many convolutions were atrophied. The cerebral substance was hyperæmic and somewhat softened. The lesions of other organs were insignificant. The stomach presented evidences of chronic catarrh —*Progès Medical.*

FERRAN, who, a year ago, attained a world-wide distinction from his so-called preventive inoculations for cholera, has recently published an article on "Gout and its Treatment," which is attracting considerable attention in the Spanish medical journals. The author calls attention to the injurious effect of the salts of soda upon those predisposed to gout. He observes that the disease is most frequent among the inhabitants of maritime cities, and is common among the passengers of vessels carrying salt. Alkaline waters also precipitate the attacks, which are followed by periods of alleviation. Whisky and other alcoholic liquors, excite attacks, which appear to cure the patient, for the time being. For treatment he recommends the acetic extract of colchicum and inhalation of nitrite of amyl, which latter drug diminishes arterial tension and facilitates the elimination of uric acid. Nitrite of amyl is an excellent remedy in those cases which will not bear the nauseating and depressant action of colchicum.—*Revist. de Cienciàs Medicas.*

---

FOR the treatment of leucorrhœa and fœtid vaginal discharges, *La Rev. des Mal. des Femmes* recommends the following:

| | | |
|---|---|---|
| ℞ | Potassii Chloratis. - - - | ʒiii. |
| | Tinct. Opii - - - - | ʒiii |
| | Aq. Picis Liq., - - - | ʒviii. |

M. Sig. Two or three teaspoonfuls to a quart of warm water, injected morning and evening. When there is pruritus vulvæ with the leucorrhœa, a solution of equal parts of tincture of iodine and iodide of potassium should be made, and a teaspoonful of this in one or two quarts of hot tar-water injected twice a day.

---

TEST FOR BILE IN URINE.—A writer in the *National Druggist*, direct attention to chloroform as a test for bile in the urine. It is ready, delicate and certain. All that is necessary is to agitate a few drops of it in a test-tube, along with the suspected urine. If bile be present, the chloroform becomes turbid, and acquires a yellowish hue, the depth of which is in proportion to the amount of bile present, in the urine. If no bile be present, the test-fluid remains limpid.—*American Medical Druggist.*

---

THE fortieth anniversary of Dubois Reymond's connection with the University of Berlin was celebrated in July. He was a student of the distiguished Müller, and among his fellow pupils were Reichert, Schwann, Virchow, Helmholtz, Henle, Haeckel and Max Schultze. He is, to-day, the world's greatest physiologist, having done more than any other man to clear up the physiology of muscles and nerves, besides contributing to other branches of this science.

---

A PATIENT of Pasteur's, bitten in the hand by a dog on the 21st of April, and treated in Paris from the 4th to the 13th of May, died July 21st of rabies. An autopsy was made and the medulla sent to Pasteur. A rabbit and a dog were inoculated from this and both died. Pasteur states that this is the first time that his treatment has been unsuccessful in a patient bitten on the hand.

---

DR. M. CHARRIN, of Paris, has discovered that cataract may be produced in rabbits by the internal administration of naphthaline. The disease has thus been induced in five rabbits, by the daily administration of from nine to twelve minims of the drug, to each pound of the animal. These doses are fifteen times greater than the ordinary dose for man.

---

THE Board of Health of Buffalo is about to enforce the law, recently made, requiring the licensing of "baby farms." This industry will in the future be placed under the auspices of the sanitary police, and it is to be hoped infanticide from want of care and starvation, now so common in "baby farms," will be suppressed.

---

PROF. GUISEPPE SORMANI, after carefully trying Cantani's method of treating phthisis by inhalations of the *bacterium termo*, concludes that it is entirely destitute of curative properties. Equally good results were obtained by using dry sterilized bread powder.

---

DR. M. SEE employs a 10 per cent. solution of chloral for injection into the tunica vaginalis in the treatment of hydrocele. This solution, he claims, has all the advantages and none of the the objections of tincture of iodine.

---

M. ELISEFF presented to the French Anthropological Society a woman with a caudal appendage covered with hair. This anomaly was present in several of the maternal ancestors of the woman.

Duclaux has studied the action of sunlight upon chemical transformations. He finds that the effects of light on nutritive liquids is analagous to that of microbes on the same substances.

---

The Brazilian Government has sent a commission to Paris to study the methods of Pasteur for the prevention of rabies.

---

Dr. S. T. Howell has been appointed lecturer on pathology, in the medical department of the University of Buffalo.

